# Impact of Glucose Loading on Variations in CD4^+^ and CD8^+^ T Cells in Japanese Participants with or without Type 2 Diabetes

**DOI:** 10.3389/fendo.2018.00081

**Published:** 2018-03-20

**Authors:** Aika Miya, Akinobu Nakamura, Hideaki Miyoshi, Yoshinari Takano, Kana Sunagoya, Koji Hayasaka, Chikara Shimizu, Yasuo Terauchi, Tatsuya Atsumi

**Affiliations:** ^1^Department of Rheumatology, Endocrinology and Nephrology, Faculty of Medicine and Graduate School of Medicine, Hokkaido University, Sapporo, Japan; ^2^Division of Laboratory and Transfusion Medicine, Hokkaido University Hospital, Sapporo, Japan; ^3^Department of Endocrinology and Metabolism, Graduate School of Medicine, Yokohama City University, Yokohama, Japan

**Keywords:** proportion of CD4^+^ and CD8^+^ T cells, oral glucose tolerance test, glucose loading, glucose metabolism, lymphocytes

## Abstract

**Objective:**

The aim of this study was to examine the fluctuations in CD4^+^ T cells, CD8^+^ T cells, and natural CD4^+^CD25^+^FoxP3^+^T-regulatory (Treg) cells following an oral glucose tolerance test (OGTT) in participants with and those without type 2 diabetes (T2DM).

**Methods:**

19 Japanese participants with T2DM (DM group) and 21 participants without diabetes (non-DM group) were recruited and underwent a 75-g OGTT. The cell numbers of leukocytes, lymphocytes, and the T cell compartment, such as CD4^+^, CD8^+^, and Treg, were calculated for blood samples obtained after an overnight 12 h fast and during a 75-g OGTT at 60 and 120 min.

**Results:**

Before glucose loading, no differences in the cell numbers of leukocytes, lymphocytes, CD4^+^, CD8^+^, and Treg were observed between the DM group and the non-DM group. The proportion of CD8^+^ was significantly reduced, whereas the proportion of CD4^+^ was significantly increased, after 120 min of glucose loading in both groups. The proportion of Treg was not affected. Furthermore, a significant positive correlation was observed between the AUC_0–120 min_ of CD8^+^ and the change in the free fatty acid level following the OGTT (ρ = 0.39, *P* < 0.05), but not that of glucose or insulin.

**Conclusion:**

The proportion of CD4^+^ T cells was increased and that of CD8^+^ T cells was reduced after glucose loading in both subjects with and without diabetes. These findings suggest that glucose loading dynamically affects the balance of the circulating T lymphocyte subset, regardless of glucose tolerance.

## Introduction

Type 2 diabetes (T2DM) is characterized by two major features: peripheral insulin resistance and impaired insulin secretion from pancreatic beta cells. Since obesity leads to the enhancement of insulin resistance, the morbidity rate for T2DM has increased with the increase in the prevalence of obesity (1, 2). The pathogenesis of obesity and T2DM involves chronic inflammation in obese adipose tissue through the acceleration of insulin resistance (3, 4). Obese adipose tissue causes inflammatory macrophages to infiltrate the visceral adipose tissue (4), promoting the migration of not only proinflammatory macrophages, but also peripheral blood T cells, thereby exacerbating chronic inflammation (5).

The peripheral blood T cell subset as well as the total peripheral leukocyte number is associated with obesity and diabetes (6, 7). In morbidly obese (body mass index [BMI] >40 kg/m^2^) participants without T2DM, the peripheral blood CD4^+^ T cell number and subset both increased. Furthermore, the CD4^+^ T cell number was correlated with the fasting insulin level regardless of the blood glucose level (6). The absolute numbers of leukocytes and T cells in the circulation were significantly enhanced in obese participants with T2DM, compared with obese participants without T2DM (7).

However, these blood samples reflect the steady state, such as after an overnight fast. Thus, little is known about the effect of acute changes in glucose metabolism induced by glucose loading on the peripheral blood T cell subset. This study was conducted to examine the fluctuations of CD4^+^, CD8^+^, and T-regulatory (Treg) cells following a 75-g oral glucose tolerance test (OGTT) in Japanese subjects with or without T2DM. To assess the relationship with T cell fluctuation, we measured the variations in glucose, insulin, and free fatty acid (FFA) levels caused by the OGTT.

## Materials and Methods

### Participants

Nineteen Japanese participants with T2DM (DM group) and 21 without diabetes (non-DM group) over 20 years of age who were treated at Hokkaido University Hospital between 2014 and 2015 were recruited for this study. All the participants were admitted to the hospital for endocrinological examinations. The exclusion criteria were type 1 diabetes and participants receiving insulin therapy. Other exclusion criteria were participants with diabetic ketosis or coma, serious infection, pregnancy or lactation, and participants scheduled to undergo surgery. Participants were also excluded if they were receiving medications that could influence glucose metabolism, such as glucocorticoids, growth hormone treatment, and immunosuppressants. The protocol for this research was reviewed by the institutional review board of Hokkaido University Hospital (013-0083) and conformed to the provisions of the Declaration of Helsinki. Signed informed consent was obtained from all the participants. All the evaluations were performed at Hokkaido University Hospital. The clinical examination consisted of a medical history, physical examination, and anthropometric measurements.

### Clinical and Laboratory Evaluation

The weight and height of the participants were measured using a calibrated scale after they had removed their shoes and any heavy clothing. The plasma glucose and serum insulin levels were measured after an overnight 12-h fast and during a 75-g OGTT at 30, 60, 90, and 120 min. The serum FFA levels and cell numbers of leukocytes, lymphocytes, and T cell subsets, such as CD4^+^, CD8^+^, and Treg were calculated for blood samples obtained after an overnight 12-h fast and during a 75-g OGTT at 60 and 120 min.

The plasma glucose level was measured using an automated glucose analyzer (GA 08 II; A&T Corporation) based on the GOD immobilized 02 electrode method. The serum insulin level was measured using the E-test TOSOH II (IRI) (Tosoh Corporation) based on a fluorescence-enzyme immunoassay. The total leukocyte count was measured using the XE-5000 automated hematology analyzer (Sysmex), the FFA was measured using NEFA-SS “EIKEN” based on an enzymatic method, and the other biochemical parameters were measured using a conventional automated analyzer between January 2014 and March 2015 at Hokkaido University Hospital. The leukocyte subpopulations were determined using flow cytometry with fluorescent monoclonal antibodies for CD4, CD8, and CD25 (Beckman Coulter) and the Human Regulatory T Cell Whole Blood Staining Kit (eBioscience).

Body mass index was calculated as the weight in kilograms divided by the height in meters squared. Participants were regarded as having a diagnosis of diabetes if they had a history of diabetes and were receiving oral hypoglycemic agents or had either a plasma HbA1c value of more than 48 mmol/mol (6.5%), a fasting plasma glucose (FPG) level of >7 mmol/L, or a 120-min value of >11.1 mmol/L in a 75-g OGTT (8). Insulin sensitivity was estimated using the homeostasis model assessment of insulin resistance (HOMA-IR), calculated as [fasting plasma insulin (μU/mL) × FPG (mmol/L)]/22.5. The homeostasis model assessment of β-cell function (HOMA-β) was calculated using the following formula: 20 × fasting insulin (μIU/mL)/fasting glucose (mmol/mL)/3.5 (9). The adipocyte insulin resistance index (adipocyte IR index) was calculated as fasting serum FFA (μEq/L) × fasting serum insulin (μU/mL) × 10^−3^ (10). Glucose-stimulated insulin secretion was evaluated based on the insulinogenic index (Δinsulin [0–30 min]/Δglucose [0–30 min]), which estimates the early-phase of insulin secretion based on an OGTT (11). For the CD4^+^ and CD8^+^ parameters, the area under the concentration-time curve for 0–120 min (AUC_0–120 min_) was calculated using the trapezoid rule.

### Statistical Analysis

The results were expressed as the means ± SD. Differences between two groups were analyzed for statistical significance using the Mann–Whitney *U* test. We also used the Wilcoxon signed test to compare the parameters at 0 min and at 120 min after glucose loading. The correlation coefficients were calculated using a Spearman rank-order correlation. A *P* value <0.05 was considered statistically significant. We performed the statistical analyses using JMP 11 (SAS Institute Inc., Cary, NC, USA) and Microsoft Excel Statistics 2011 for Mac (SSRI Co. Ltd., Tokyo, Japan).

## Results

Nineteen Japanese participants in the DM group and 21 in the non-DM group were enrolled in this study. Among the DM group, 13 were treated without hypoglycemic medication; the remainder were treated with a dipeptidyl peptidase 4 (DPP-4) inhibitor (*n* = 2), a biguanide (*n* = 1), a glucagon-like peptide-1 receptor agonist (*n* = 1), a combination of sulfonylurea and a biguanide (*n* = 1), a combination of a DPP-4 inhibitor and a biguanide (*n* = 1), or a triple-drug combination of sulfonylurea, a biguanide, and a DPP-4 inhibitor (*n* = 1). The baseline clinical and metabolic characteristics of both groups are shown in Table 1. As expected, the participant age, HbA1c level, and FPG level were significantly higher in the DM group than in the non-DM group. Meanwhile, the insulinogenic index in the DM group was significantly lower than that in the non-DM group. No statistically significant differences in sex, BMI, fasting serum insulin, total cholesterol, triglycerides, low-density lipoprotein cholesterol, high-density lipoprotein cholesterol, or FFA levels or the HOMA-IR or adipocyte IR index were observed between the two groups. These results indicated that the participants in the DM group were not in a hyperinsulinemic state, although they did have impaired insulin secretion. As shown in Table 2, the mean numbers of leukocytes and lymphocytes before glucose loading were comparable between the two groups. Detailed flow cytometric analyses revealed no differences in the CD4^+^, CD8^+^, or Treg cell numbers or the CD4^+^/CD8^+^ or Treg/CD4^+^ ratios between the two groups.

**Table 1:** Baseline characteristics of the total, participants with type 2 diabetes (DM), and participants without diabetes (non-DM) groups.

	Total	DM	Non-DM	*P* value
*n*	40	19	21	
Age (years)	55.0 ± 14.3	61.6 ± 13.1	49.0 ± 12.8	<0.01
Female sex (%)	57.5	52.6	61.9	0.55
BMI (kg/m^2^)	25.8 ± 6.1	26.1 ± 6.8	25.5 ± 5.6	0.82
HbA1c (mmol/mol)	43.8 ± 11.6	50.6 ± 13.5	37.6 ± 4.0	<0.01
HbA1c (%)	6.2 ± 1.1	6.8 ± 1.2	5.6 ± 0.4	<0.01
FPG (mmol/L)	5.9 ± 1.8	6.6 ± 2.4	5.2 ± 0.5	<0.01
FPI (μU/mL)	5.5 ± 3.8	5.7 ± 4.1	5.4 ± 3.7	0.81
Total cholesterol (mg/dL)	193.4 ± 32.7	182.0 ± 31.9	203.6 ± 30.7	0.05
Triglyceride (mg/dL)	135.9 ± 88.3	133.7 ± 100.8	137.8 ± 77.9	0.34
HDL cholesterol (mg/dL)	54.6 ± 15.5	52.6 ± 16.5	56.4 ± 14.6	0.32
LDL cholesterol (mg/dL)	120.8 ± 31.4	112.1 ± 36.3	128.7 ± 24.4	0.06
Free fatty acid (μEq/L)	672.5 ± 381.0	679.0 ± 298.8	666.6 ± 450.2	0.32
HOMA-IR	1.5 ± 1.3	1.8 ± 1.6	1.3 ± 0.9	0.49
Homeostasis model assessment of β-cell function	1.1 ± 0.8	1.0 ± 0.8	1.2 ± 0.8	0.56
Insulinogenic Index	9.0 ± 9.7	5.2 ± 3.8	12.4 ± 12.0	<0.01
Adipocyte IR index	4.4 ± 5.3	4.5 ± 4.3	4.2 ± 6.2	0.47

*Values are the mean ± SD*.

*BMI, body mass index; FPG, fasting plasma glucose; FPI, fasting plasma insulin; HOMA-IR, homeostasis model assessment of insulin resistance; adipocyte IR index, adipocyte insulin resistance index*.

**Table 2:** Absolute numbers of leukocyte subpopulations in peripheral blood of participants before glucose loading.

	Total	DM	Non-DM	*P* value
Leukocytes (/μL)	6292.3 ± 1990.0	6466.7 ± 2332.9	6142.9 ± 1687.5	0.96
Lymphocytes (/μL)	2159.3 ± 954.7	2001.2 ± 636.7	2294.8 ± 1159.9	0.78
CD4^+^ (/μL)	963.5 ± 369.3	896.5 ± 290.9	1020.9 ± 423.9	0.38
CD8^+^ (/μL)	598.6 ± 279.5	582.7 ± 277.8	612.2 ± 287.1	0.80
Treg (/μL)	83.9 ± 38.5	78.6 ± 38.1	88.6 ± 39.2	0.32
CD4^+^/CD8^+^	1.8 ± 0.6	1.7 ± 0.6	1.8 ± 0.6	0.91
Treg/CD4^+^	0.08 ± 0.02	0.09 ± 0.03	0.09 ± 0.02	0.70

*Values are the mean ± SD*.

**Figure 1:**
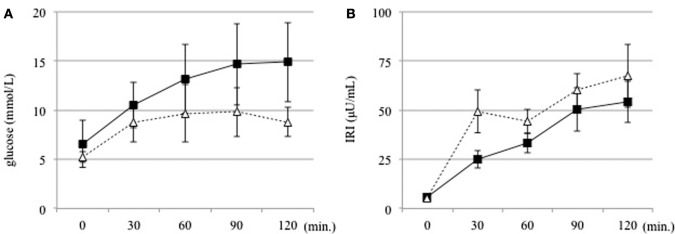
**(A)** Serum glucose and **(B)** insulin levels were measured at 30 min intervals before and for 120 min after an oral glucose tolerance test in the DM (solid line) and non-DM (dotted line) groups.

**Figure 2:**
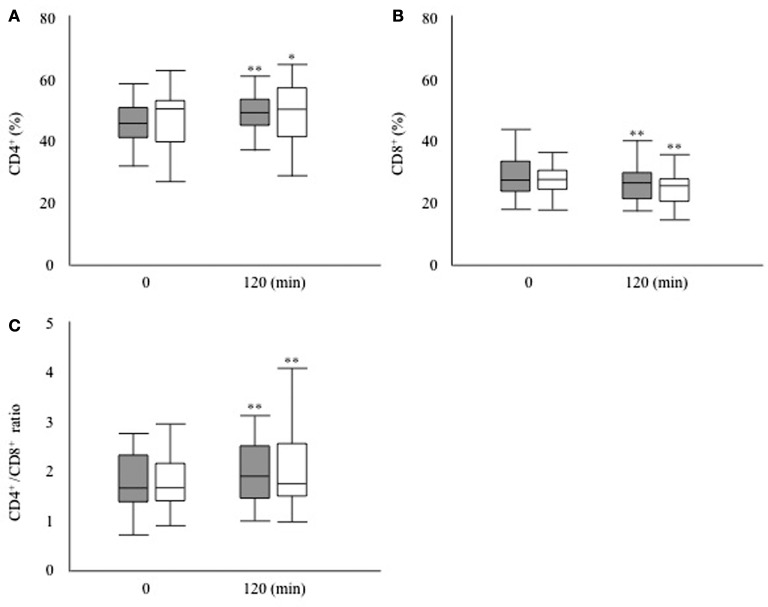
**(A)** Proportion of CD4^+^ T cells, **(B)** proportion of CD8^+^ T cells, and **(C)** CD4^+^/CD8^+^ ratio after 120 min of glucose loading during an oral glucose tolerance test in the participants with type 2 diabetes (filled bar) and the participants without diabetes (open bar). *P < 0.05 vs. 0 min, **P < 0.01 vs. 0 min.

Next, we investigated whether the T cell subset fluctuates after glucose loading during an OGTT (Figure 1). The absolute numbers of leukocytes had decreased significantly after 120 min of glucose loading in both groups (DM group, 6,466.7 ± 2,332.9 to 6066.7 ± 2262.5/μL, *P* < 0.01; non-DM group, 6,142.9 ± 1,687.5 to 5919.0 ± 1546.2/μL, *P* < 0.05). The proportion of lymphocytes was not modified after 120 min of glucose loading in both groups (DM group, 31.8 ± 7.3 to 30.2 ± 7.4%, *P* = 0.09; non-DM group, 37.9 ± 15.7 to 37.2 ± 15.2%, *P* = 0.07). Within the T cell subset, the proportion of CD4^+^ cells increased significantly, as shown in Figure 2A, whereas the proportion of CD8^+^ cells decreased significantly (Figure 2B) at 120 min after glucose loading in both groups. Likewise, a significant increase in the CD4^+^/CD8^+^ ratio, which fluctuates together with the variations in CD4^+^ and CD8^+^ T cell numbers, was observed after glucose loading in both groups (Figure 2C). The proportion of Treg cells were not modified at 120 min after glucose loading in both groups (DM group, 78.6 ± 38.1 to 81.0 ± 45.8/μL, *P* = 0.07; non-DM group, 88.6 ± 39.2 to 86.1 ± 36.7/μL, *P* = 0.47). The Treg/CD4^+^ ratio were not different after 120 min of glucose loading in both groups (DM group, 0.09 ± 0.03 to 0.09 ± 0.03; *P* = 0.07, and non-DM group, 0.09 ± 0.02 to 0.09 ± 0.02; *P* = 0.47). No significant differences in any of these absolute numbers or the proportions at 0 or 120 min were observed between the DM group and the non-DM group.

To reveal the factors influencing the fluctuation of T cell subsets, we examined the relations between the fluctuations in the T cell subsets and the parameters reflecting insulin sensitivity and insulin secretion in all the participants. As shown in Table 3, these parameters were not correlated with the AUC_0–120 min_ of CD4^+^ or CD8^+^ during the OGTT.

**Table 3:** Correlations between fluctuations in T cells and parameters reflecting insulin sensitivity and insulin secretion.

	AUC_0–120 min_ of CD4^**+**^(×10^3^/μL × min)		AUC_0–120 min_ of CD8^**+**^(×10^3^/μL × min)	
	
	ρ	*P* value	ρ	*P* value
Body mass index (kg/m^2^)	0.22	0.17	0.20	0.23
the homeostasis model assessment of insulin resistance	0.24	0.14	0.12	0.48
Homeostasis model assessment of β-cell function	0.19	0.24	0.09	0.59
Insulinogenic Index	−0.03	0.86	0.08	0.65
Adipocyte insulin resistance index	0.21	0.19	0.09	0.59

**Figure 3:**
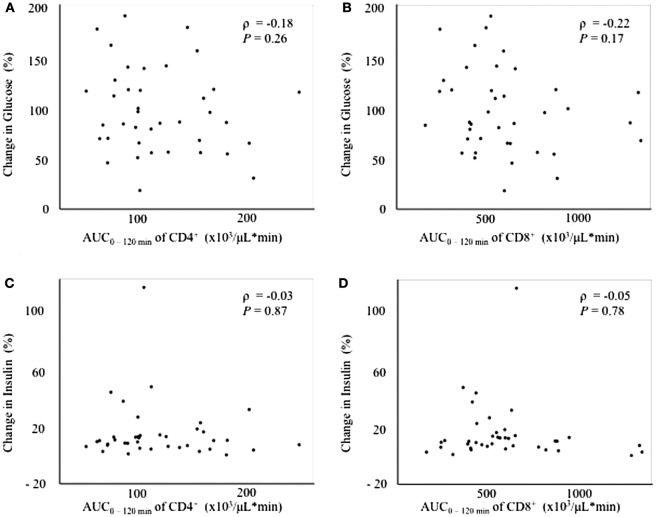
Correlations between changes in glucose or insulin levels and fluctuations in peripheral blood CD4^+^ or CD8^+^ T cells. **(A)** Correlation between the change in glucose_0–120 min_ and the AUC_0–120 min_ of CD4^+^. **(B)** Correlation between the change in glucose_0–120 min_ and the AUC_0–-120 min_ of CD8^+^. **(C)** Correlation between the change in insulin_0–120 min_ and the AUC_0–120 min_ of CD4^+^. **(D)** Correlation between the change in insulin_0–120 min_ and the AUC_0–120 min_ of CD8^+^.

**Figure 4:**
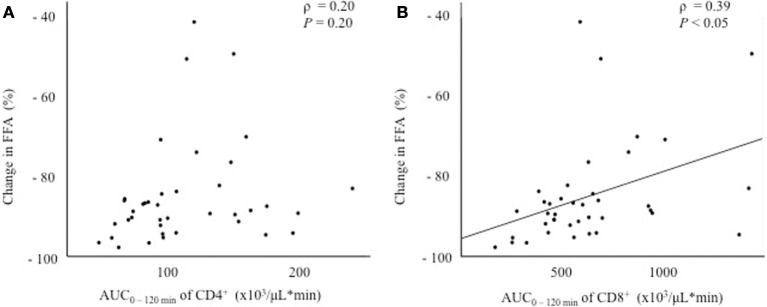
Correlations between the change in the free fatty acid (FFA) level and fluctuations in peripheral blood CD4^+^ or CD8^+^ T cells. **(A)** Correlation between the change in FFA_0–120 min_ and the AUC_0–120 min_ of CD4^+^. **(B)** Correlation between the change in FFA_0–120 min_ and the AUC_0–120 min_ of CD8^+^.

Since the fluctuation in glucose metabolism caused by the OGTT could affect the fluctuation of the T cell subset, we performed additional correlation analyses by calculating the change in the plasma glucose, serum insulin, and FFA levels during the OGTT. As a matter of course, the glucose and insulin levels increased significantly after glucose loading, whereas the FFA levels decreased significantly after glucose loading during the OGTT (data not shown). Although no correlations were found between the change in glucose or insulin levels and the AUC_0–120 min_ of CD4^+^ or CD8^+^ (Figure 3), a significant positive correlation was observed between the change in the FFA level and the AUC_0–120 min_ of CD8^+^ (ρ = 0.39, *P* < 0.05), but not of CD4^+^, following the OGTT (Figure 4).

## Discussion

This study reported alterations in the peripheral T cell subset following glucose loading in both subjects with and those without diabetes. The main findings of our study were the following: (1) the proportion of CD4^+^ T cells increased and that of CD8^+^ T cells decreased after glucose loading during a 75-g OGTT in both participants with and those without diabetes, and (2) the fluctuation of CD8^+^ was associated with that of FFA after glucose loading, but not the elevation of the glucose and insulin levels. Thus, glucose loading can change the balance of the peripheral T cell subset regardless of glucose tolerance.

As reported in morbidly obese participants under a steady state condition, the increased peripheral blood CD4^+^ T cell number might result from increased CD4^+^ T cell proliferation (6). However, few studies have focused on the effect of acute changes in glucose metabolism arising from glucose loading on the peripheral blood T cell subset. Although our study showed that the proportion of CD4^+^ T cells increased and that of CD8^+^ T cells decreased after glucose loading, the reason why the proportions of CD4^+^ and CD8^+^ T cells changed after glucose loading remains unclear. Several mechanisms, such as changes in thymic output, peripheral proliferation, and altered redistribution, can be considered. Since adipose tissue-derived FFA has been shown to enhance T cell proliferation (7, 12), we speculated that the fluctuation of FFA after glucose loading might have dynamically affected T cell proliferation and the balance of the circulating T cell subset. Therefore, a future challenge will be to elucidate the mechanism of the changes in the proportions of CD4^+^ and CD8^+^ T cells.

Regarding the association between the changes in the T cell subset and insulin resistance, a significant positive correlation was observed between the AUC_0–120 min_ of CD8^+^ and the change in the FFA level, but not the glucose or insulin levels, after glucose loading (Figures 3 and 4). In other words, the decrease in CD8^+^ T cells was associated with a decrease in the FFA level after glucose loading. FFA is mobilized from triglycerides, stored in adipose tissue through the process of lipolysis (13). Insulin secretion after glucose loading inhibits lipolysis in adipose tissue (14). Under the condition of insulin resistance in adipose tissue, insulin is unable to suppress lipolysis, resulting in a lack of FFA suppression after glucose loading. Our results suggest that an inadequate decrease in CD8^+^ T cells after glucose loading might reflect insulin resistance in adipose tissues. However, no correlation was found between insulin resistance in adipose tissue estimated according to the adipocyte IR index and the change in the AUC_0–120 min_ of CD8^+^ (Table 3). The adipocyte IR index is not an appropriate parameter for examining the acute changes in glucose metabolism that occur following glucose loading because the adipocyte IR index is calculated using fasting plasma FFA and insulin levels, but not parameters that are examined after glucose loading. Further additional consideration is needed to elucidate the association between the fluctuations of FFA levels and CD8^+^ T cells. The association between the changes in the proportion of the T cell subset and the FFA levels prompted us to examine the association between the changes in the proportion of the T cell subset and other serum lipid profiles. As a result, the total cholesterol, triglycerides, low-density lipoprotein cholesterol, and high-density lipoprotein cholesterol levels were found not to be correlated with the changes in the proportion of CD4^+^ or CD8^+^ during the OGTT (Tables S3–S5 and S8).

As suggested in several reports, DPP-4 inhibitors, biguanide, cholesterol-lowering agents, angiotensin II receptor blockers (ARB), and angiotensin-converting enzyme inhibitors could cause changes in the proportion of T lymphocytes (15–19). We, therefore, performed a sub-analysis divided according to each medication. In the DM group, four participants were treated with a DPP-4 inhibitor. Similar baseline characteristics and changes in the proportion of the T cell subset during an OGTT were observed between the 15 subjects who were not taking a DPP-4 inhibitor in the DM group and the 19 subjects in the DM group (Tables S1 and S2). In the DM group, four participants were treated with biguanide. Similar baseline characteristics and changes in the proportion of the T cell subset during an OGTT were observed in the 15 subjects who were not taking biguanide in the DM group and the 19 subjects in the DM group (Tables S9 and S10). In the DM and non-DM groups, five participants in each group were treated with statin. And one patient was treated with fibrate and one patient was treated with ezetimibe in the DM groups. Similar baseline characteristics and changes in the proportion of the T cell subset during an OGTT were observed in a non-cholesterol-lowering agent group (*n* = 28) of participants who were not taking cholesterol-lowering agent and a cholesterol-lowering agent group (*n* = 12) of participants who were receiving medications for dyslipidemia (Tables S6, S7, S11 and S12). Also, four participants in DM group and one patient in NDM group were treated with ARB. Similar baseline characteristics and changes in the proportion of the T cell subset during an OGTT were observed in a non-ARB group (*n* = 35) of participants who were not taking ARB and an ARB group (*n* = 5) of participants who were receiving ARB (Tables S13–S15). We suggest that the use of medication had no practical impact on our results.

A previous study has already reported that the proportion of T cells was changed after glucose loading in subjects without diabetes (20). Our study reported alterations in the T cell subset in both subjects with and those without diabetes. Furthermore, this study found that the changes in the proportion of the T cell subset were associated with the changes in FFA after glucose loading.

This study had some limitations. Our study had a relatively small sample and the mechanism responsible for the changes in the proportion of CD4^+^ and CD8^+^ T cells is presently unclear. Also, since most of the participants in our study were Japanese, whether our results are applicable to non-Japanese participants remains unclear. Ethnic differences in the pathophysiological mechanisms of diabetes, such as the degree of obesity, the insulin secretion capacity, and insulin resistance, have been documented between Japanese and Western participants.

We found that the proportion of CD4^+^ T cells increased and that of CD8^+^ T cells decreased after glucose loading in subjects with and those without diabetes. These findings suggest that glucose loading dynamically affects the balance of circulating T lymphocyte subsets, regardless of glucose tolerance.

## Ethics Statement

The protocol for this research was reviewed by the institutional review board of Hokkaido University Hospital (013-0083) and conformed to the provisions of the Declaration of Helsinki. Signed informed consent was obtained from all the participants.

## Author Contributions

KS and KH analyzed the patient data. AM and AN were major contributors in writing the manuscript. HM, YoT, CS, YaT and TA read and approved the final manuscript.

## Conflict of Interest Statement

The authors declare that the research was conducted in the absence of any commercial or financial relationships that could be construed as a potential conflict of interest. The reviewer SO and handling Editor declared their shared affiliation.

## Abbreviations

T2DM, type 2 diabetes; BMI, body mass index; Treg, T-regulatory cells; OGTT, oral glucose tolerance test; FFA, free fatty acid; DM group, participants with type 2 diabetes; non-DM group, participants without diabetes; HOMA-IR, the homeostasis model assessment of insulin resistance; adipocyte IR index, adipocyte insulin resistance index; DPP-4, dipeptidyl peptidase 4.
