# Supplementary material for: Impact of Glucose Loading on Variations in CD4+ and CD8+ T Cells in Japanese Participants with or without Type 2 Diabetes
Source: Front Endocrinol (Lausanne). 2018 Mar 20;9:81. doi: 10.3389/fendo.2018.00081 (PMC5870166; doi:10.3389/fendo.2018.00081)
Supplement: Supplementary file 1 [file table_1.doc]

Table s1. Baseline characteristics of the DM group and the DM group without DPP-4 inhibitors

|  | DM group | DM group without DPP-4 inhibitors | *P* value |
| --- | --- | --- | --- |
| n | 19 | 15 |  |
| Age (years) | 61.6 ± 13.1 | 62.7 ± 12.9 | 0.81 |
| Female sex (%) | 52.6 | 53.3 | 0.97 |
| BMI (kg/m^2^) | 26.1 ± 6.8 | 26.4 ± 7.1 | 0.92 |
| HbA1c (mmol/mol) | 50.6 ± 13.4 | 46.9 ± 7.9 | 0.42 |
| HbA1c (%) | 6.8 ± 1.2 | 6.4 ± 0.8 | 0.42 |
| FPG (mmol/L) | 6.6 ± 2.4 | 6.0 ± 0.9 | 0.68 |
| FPI (μU/mL) | 5.7 ± 4.1 | 5.6 ± 4.4 | 0.93 |
| Free fatty acid (μEq/L) | 679.0 ± 298.8 | 610.2 ± 212.9 | 0.60 |
| HOMA-IR | 1.8 ± 1.6 | 1.5 ± 1.2 | 0.84 |
| HOMA-β | 1.0 ± 0.8 | 1.1 ± 0.9 | 0.88 |
| Insulinogenic Index | 5.2 ± 3.8 | 5.3 ± 4.0 | 0.94 |
| Adipocyte IR index | 4.5 ± 4.3 | 4.0 ± 4.3 | 0.70 |

Values are the mean ± S.D.
