# Supplementary material for: Impact of Glucose Loading on Variations in CD4+ and CD8+ T Cells in Japanese Participants with or without Type 2 Diabetes
Source: Front Endocrinol (Lausanne). 2018 Mar 20;9:81. doi: 10.3389/fendo.2018.00081 (PMC5870166; doi:10.3389/fendo.2018.00081)
Supplement: Supplementary file 2 [file table_2.doc]

Table s2. Changes in the proportion of the T cell subset at 120 min after glucose loading during an OGTT in the DM group

|  | DM group | DM group without DPP-4 inhibitors | *P* value |
| --- | --- | --- | --- |
| CD4^+^ (%) | 2.40 ± 3.56 | 2.59 ± 3.90 | 0.73 |
| CD8^+^ (%) | -2.04 ± 3.24 | -2.44 ± 3.37 | 0.76 |
| Treg (%) | 0.55 ± 2.22 | 0.22 ± 2.20 | 0.73 |
| CD4^+^/CD8^+^ | 0.21 ± 0.23 | 0.24 ± 0.23 | 0.68 |
| Treg/CD4^+^ | 0.005 ± 0.022 | 0.002 ± 0.022 | 0.73 |

Values are the mean ± S.D.
