# Supplementary material for: Impact of Glucose Loading on Variations in CD4+ and CD8+ T Cells in Japanese Participants with or without Type 2 Diabetes
Source: Front Endocrinol (Lausanne). 2018 Mar 20;9:81. doi: 10.3389/fendo.2018.00081 (PMC5870166; doi:10.3389/fendo.2018.00081)
Supplement: Supplementary file 3 [file table_3.doc]

Table s3. Changes in the proportion of the T cell subset at 120 min after glucose loading during an OGTT in the DM group

|  | High adipo IR | Low adipo IR | *P* value |
| --- | --- | --- | --- |
| CD4^+^ (%) | 1.65 ± 1.55 | 3.34 ± 5.07 | 0.62 |
| CD8^+^ (%) | -1.78 ± 1.33 | -2.38 ± 4.80 | 0.89 |
| Treg (%) | 0.82 ± 2.18 | 0.21 ± 2.36 | 0.86 |
| CD4^+^/CD8^+^ | 0.18 ± 0.13 | 0.24 ± 0.32 | 0.66 |
| Treg/CD4^+^ | 0.008 ± 0.022 | 0.002 ± 0.024 | 0.86 |

Values are the mean ± S.D.
