# Supplementary material for: Impact of Glucose Loading on Variations in CD4+ and CD8+ T Cells in Japanese Participants with or without Type 2 Diabetes
Source: Front Endocrinol (Lausanne). 2018 Mar 20;9:81. doi: 10.3389/fendo.2018.00081 (PMC5870166; doi:10.3389/fendo.2018.00081)
Supplement: Supplementary file 4 [file table_4.doc]

Table s4. Changes in the proportion of the T cell subset at 120 min after glucose loading during an OGTT in the DM group

|  | High BMI | Low BMI | *P* value |
| --- | --- | --- | --- |
| CD4^+^ (%) | 2.24 ± 4.86 | 2.56 ± 1.77 | 0.13 |
| CD8^+^ (%) | -2.43 ± 4.19 | -1.66 ± 2.12 | 0.72 |
| Treg (%) | 0.58 ± 2.23 | 0.51 ± 2.35 | 0.51 |
| CD4^+^/CD8^+^ | 0.19 ± 0.27 | 0.23 ± 0.19 | 0.35 |
| Treg/CD4^+^ | 0.006 ± 0.022 | 0.005 ± 0.023 | 0.54 |

Values are the mean ± S.D.
