# Supplementary material for: Impact of Glucose Loading on Variations in CD4+ and CD8+ T Cells in Japanese Participants with or without Type 2 Diabetes
Source: Front Endocrinol (Lausanne). 2018 Mar 20;9:81. doi: 10.3389/fendo.2018.00081 (PMC5870166; doi:10.3389/fendo.2018.00081)
Supplement: Supplementary file 5 [file table_5.doc]

Table s5. Changes in the proportion of the T cell subset at 120 min after glucose loading during an OGTT in the DM group

|  | High HOMA-R | Low HOMA-R | *P* value |
| --- | --- | --- | --- |
| CD4^+^ (%) | 0.68 ± 1.00 | 3.06 ± 3.98 | 0.06 |
| CD8^+^ (%) | -1.62 ± 0.72 | -2.21 ± 3.82 | 0.62 |
| Treg (%) | 0.34 ± 2.90 | 0.62 ± 2.04 | 0.40 |
| CD4^+^/CD8^+^ | 0.16 ± 0.10 | 0.22 ± 0.26 | 0.52 |
| Treg/CD4^+^ | 0.003 ± 0.029 | 0.006 ± 0.020 | 0.40 |

Values are the mean ± S.D.
