# Supplementary material for: Impact of Glucose Loading on Variations in CD4+ and CD8+ T Cells in Japanese Participants with or without Type 2 Diabetes
Source: Front Endocrinol (Lausanne). 2018 Mar 20;9:81. doi: 10.3389/fendo.2018.00081 (PMC5870166; doi:10.3389/fendo.2018.00081)
Supplement: Supplementary file 6 [file table_6.doc]

Table s6. Baseline characteristics of the statin and non-statin groups

|  | Statin | Non-statin | *P* value |
| --- | --- | --- | --- |
| n | 10 | 30 |  |
| Age (years) | 58.6 ± 13.5 | 53.8 ± 14.6 | 0.40 |
| Female sex (%) | 60.0 | 56.7 | 0.70 |
| BMI (kg/m^2^) | 25.2 ± 4.6 | 26.0 ± 6.5 | 0.98 |
| HbA1c (mmol/mol) | 47.4 ± 19.0 | 42.5 ± 8.0 | 0.63 |
| HbA1c (%) | 6.5 ± 1.7 | 6.0 ± 0.7 | 0.63 |
| FPG (mmol/L) | 6.7 ± 3.2 | 6.0 ± 0.9 | 0.29 |
| FPI (μU/mL) | 6.2 ± 4.3 | 5.3 ± 3.7 | 0.60 |
| Free fatty acid (μEq/L) | 892.6 ± 597.3 | 599.1 ± 249.4 | 0.53 |
| Total cholesterol | 187.8 ± 31.5 | 195.2 ± 33.4 | 0.63 |
| Triglyceride | 167.8 ± 127.2 | 125.2 ± 70.7 | 0.38 |
| HDL cholesterol | 57.1 ± 14.0 | 53.7 ± 16.1 | 0.36 |
| LDL cholesterol | 106.8 ± 31.0 | 125.5 ± 30.6 | 0.15 |
| HOMA-IR | 2.0 ± 2.0 | 1.3 ± 1.0 | 0.45 |
| HOMA-β | 1.1 ± 0.8 | 1.1 ± 0.8 | 0.98 |
| Insulinogenic Index | 11.0 ± 14.5 | 8.3 ± 7.7 | 0.95 |
| Adipocyte IR index | 7.1 ± 8.6 | 3.4 ± 3.4 | 0.30 |

Values are the mean ± S.D.
