# Supplementary material for: Impact of Glucose Loading on Variations in CD4+ and CD8+ T Cells in Japanese Participants with or without Type 2 Diabetes
Source: Front Endocrinol (Lausanne). 2018 Mar 20;9:81. doi: 10.3389/fendo.2018.00081 (PMC5870166; doi:10.3389/fendo.2018.00081)
Supplement: Supplementary file 7 [file table_7.doc]

Table s7. Changes in the proportion of the T cell subset at 120 min after glucose loading during an OGTT in the statin and non-statin groups

|  | Statin | Non-statin | *P* value |
| --- | --- | --- | --- |
| CD4^+^ (%) | 1.46 ± 1.58 | 1.89 ± 3.32 | 0.79 |
| CD8^+^ (%) | -0.92 ± 2.06 | -2.12 ± 2.63 | 0.25 |
| Treg (%) | 0.47 ± 1.13 | 0.15 ± 2.18 | 0.63 |
| CD4^+^/CD8^+^ | 0.18 ± 0.27 | 0.23 ± 0.25 | 0.35 |
| Treg/CD4^+^ | 0.008 ± 0.012 | 0.005 ± 0.025 | 0.92 |

Values are the mean ± S.D.
