# Supplementary material for: Impact of Glucose Loading on Variations in CD4+ and CD8+ T Cells in Japanese Participants with or without Type 2 Diabetes
Source: Front Endocrinol (Lausanne). 2018 Mar 20;9:81. doi: 10.3389/fendo.2018.00081 (PMC5870166; doi:10.3389/fendo.2018.00081)
Supplement: Supplementary file 8 [file table_8.doc]

Table s8. Correlations between the changes in proportion of the T cell subset at 120 min after glucose loading during an OGTT and serum lipid profiles

|  | Changes in proportion  of CD4^+^ (%) | | Changes in proportion  of CD8^+^ (%) | |
| --- | --- | --- | --- | --- |
|  | ρ | *P* value | ρ | *P* value |
| Total cholesterol | 0.21 | 0.21 | -0.20 | 0.22 |
| Triglyceride | 0.06 | 0.72 | -0.06 | 0.70 |
| HDL cholesterol | 0.05 | 0.77 | 0.05 | 0.78 |
| LDL cholesterol | 0.25 | 0.12 | -0.25 | 0.12 |

Values are the mean ± S.D.
