# Supplementary material for: Impact of Glucose Loading on Variations in CD4+ and CD8+ T Cells in Japanese Participants with or without Type 2 Diabetes
Source: Front Endocrinol (Lausanne). 2018 Mar 20;9:81. doi: 10.3389/fendo.2018.00081 (PMC5870166; doi:10.3389/fendo.2018.00081)
Supplement: Supplementary file 9 [file table_9.doc]

Table s9. Baseline characteristics of the DM group and the DM group without biguanide

|  | DM group | DM group without biguanide | *P* value |
| --- | --- | --- | --- |
| n | 19 | 15 |  |
| Age (years) | 61.6 ± 13.1 | 62.2 ± 14.1 | 0.77 |
| Female sex (%) | 52.6 | 60.0 | 0.67 |
| BMI (kg/m^2^) | 26.1 ± 6.7 | 26.3 ± 7.2 | 1.00 |
| HbA1c (mmol/mol) | 50.3 ± 14.8 | 50.6 ± 13.4 | 0.74 |
| HbA1c (%) | 6.8 ± 1.2 | 6.8 ± 1.4 | 0.74 |
| FPG (mmol/L) | 6.6 ± 2.4 | 6.4 ± 2.6 | 0.53 |
| FPI (μU/mL) | 5.7 ± 4.1 | 6.3 ± 4.2 | 0.63 |
| Free fatty acid (μEq/L) | 679.0 ± 298.8 | 703.9 ± 305.4 | 0.78 |
| HOMA-IR | 1.8 ± 1.6 | 1.9 ± 1.7 | 0.65 |
| HOMA-β | 1.0 ± 0.8 | 1.2 ± 0.9 | 0.60 |
| Insulinogenic Index | 5.2 ± 3.8 | 6.3 ± 3.6 | 0.30 |
| Adipocyte IR index | 4.5 ± 4.3 | 5.0 ± 4.5 | 0.68 |

Values are the mean ± S.D.
