# Supplementary material for: Impact of Glucose Loading on Variations in CD4+ and CD8+ T Cells in Japanese Participants with or without Type 2 Diabetes
Source: Front Endocrinol (Lausanne). 2018 Mar 20;9:81. doi: 10.3389/fendo.2018.00081 (PMC5870166; doi:10.3389/fendo.2018.00081)
Supplement: Supplementary file 10 [file table_10.doc]

Table s10. Changes in the proportion of the T cell subset at 120 min after glucose loading during an OGTT in the DM group and the DM group without biguanide

|  | DM group | DM group without biguanide | *P* value |
| --- | --- | --- | --- |
| CD4^+^ (%) | 2.40 ± 3.56 | 2.70 ± 3.09 | 0.46 |
| CD8^+^ (%) | -2.04 ± 3.24 | -2.44 ± 3.37 | 0.76 |
| Treg (%) | 0.55 ± 2.22 | 0.03 ± 2.00 | 0.46 |
| CD4^+^/CD8^+^ | 0.21 ± 0.23 | 0.26 ± 0.21 | 0.48 |
| Treg/CD4^+^ | 0.005 ± 0.022 | 0.001 ± 0.020 | 0.46 |

Values are the mean ± S.D.
