# Supplementary material for: Impact of Glucose Loading on Variations in CD4+ and CD8+ T Cells in Japanese Participants with or without Type 2 Diabetes
Source: Front Endocrinol (Lausanne). 2018 Mar 20;9:81. doi: 10.3389/fendo.2018.00081 (PMC5870166; doi:10.3389/fendo.2018.00081)
Supplement: Supplementary file 11 [file table_11.doc]

Table s11. Baseline characteristics of the cholesterol-lowering agent and non-cholesterol-lowering agent groups

|  | Cholesterol-  lowering agent | Non-cholesterol-  lowering agent | *P* value |
| --- | --- | --- | --- |
| n | 12 | 28 |  |
| Age (years) | 58.3 ± 12.2 | 53.5 ± 15.1 | 0.42 |
| Female sex (%) | 58.3 | 57.1 | 0.94 |
| BMI (kg/m^2^) | 25.6 ± 4.3 | 25.9 ± 6.7 | 0.75 |
| HbA1c (mmol/mol) | 47.5 ± 17.2 | 42.0 ± 8.1 | 0.29 |
| HbA1c (%) | 6.5 ± 1.6 | 6.0 ± 0.7 | 0.29 |
| FPG (mmol/L) | 6.6 ± 3.0 | 5.5 ± 0.8 | 0.19 |
| FPI (μU/mL) | 6.3 ± 3.9 | 5.2 ± 3.8 | 0.35 |
| Free fatty acid (μEq/L) | 871.6 ± 549.2 | 587.2 ± 248.2 | 0.33 |
| Total cholesterol (mg/dL) | 184.7 ± 29.4 | 197.1 ± 33.8 | 0.30 |
| Triglyceride (mg/dL) | 160.3± 116.8 | 125.4 ± 73.0 | 0.34 |
| HDL cholesterol (mg/dL) | 56.5 ± 12.8 | 53.8 ± 16.6 | 0.46 |
| LDL cholesterol (mg/dL) | 105.8 ± 28.4 | 127.3 ± 30.8 | 0.06 |
| HOMA-IR | 2.0 ± 1.8 | 1.3 ± 1.0 | 0.23 |
| HOMA-β | 1.2 ± 0.8 | 1.1 ± 0.8 | 0.68 |
| Insulinogenic Index | 9.9 ± 13.5 | 8.6 ± 7.9 | 0.64 |
| Adipocyte IR index | 6.8 ± 7.9 | 3.3 ± 3.4 | 0.13 |

Values are the mean ± S.D.
