# Supplementary material for: Impact of Glucose Loading on Variations in CD4+ and CD8+ T Cells in Japanese Participants with or without Type 2 Diabetes
Source: Front Endocrinol (Lausanne). 2018 Mar 20;9:81. doi: 10.3389/fendo.2018.00081 (PMC5870166; doi:10.3389/fendo.2018.00081)
Supplement: Supplementary file 12 [file table_12.doc]

Table s12. Changes in the proportion of the T cell subset at 120 min after glucose loading during an OGTT in the Cholesterol-lowering agent and non-cholesterol-lowering agent groups

|  | Cholesterol-  lowering agent | | Non-cholesterol-  lowering agent | *P* value |
| --- | --- | --- | --- | --- |
| CD4^+^ (%) | | 1.40 ± 1.41 | 1.94 ± 3.43 | 0.66 |
| CD8^+^ (%) | | -0.70 ± 1.94 | -2.29 ± 2.64 | 0.06 |
| Treg (%) | | 0.08 ± 1.62 | 0.005 ± 2.08 | 0.33 |
| CD4^+^/CD8^+^ | | 0.15 ± 0.26 | 0.24 ± 0.25 | 0.15 |
| Treg/CD4^+^ | | 0.01 ± 0.02 | 0.01 ± 0.02 | 0.33 |

Values are the mean ± S.D.
