# Supplementary material for: Impact of Glucose Loading on Variations in CD4+ and CD8+ T Cells in Japanese Participants with or without Type 2 Diabetes
Source: Front Endocrinol (Lausanne). 2018 Mar 20;9:81. doi: 10.3389/fendo.2018.00081 (PMC5870166; doi:10.3389/fendo.2018.00081)
Supplement: Supplementary file 13 [file table_13.doc]

Table s13. Baseline characteristics of the ARB and non-ARB groups

|  | ARB | Non-ARB | *P* value |
| --- | --- | --- | --- |
| n | 5 | 35 |  |
| Age (years) | 62.2 ± 7.2 | 53.9 ± 14.8 | 0.22 |
| Female sex (%) | 20.0 | 62.9 | 0.07 |
| BMI (kg/m^2^) | 29.0 ± 9.6 | 25.3 ± 5.4 | 0.47 |
| HbA1c (mmol/mol) | 41.8 ± 5.9 | 44.0 ± 12.3 | 0.95 |
| HbA1c (%) | 6.0 ± 0.5 | 6.2 ± 1.1 | 0.95 |
| FPG (mmol/L) | 5.8 ± 0.7 | 5.9 ± 1.9 | 0.35 |
| FPI (μU/mL) | 8.7 ± 6.7 | 5.1 ± 3.2 | 0.27 |
| Free fatty acid (μEq/L) | 931.8 ± 493.4 | 635.5 ± 355.8 | 0.09 |
| Total cholesterol (mg/dL) | 189.0 ± 34.5 | 194.0 ± 33.0 | 0.81 |
| Triglyceride (mg/dL) | 101.4 ± 52.0 | 140.7 ± 91.8 | 0.30 |
| HDL cholesterol (mg/dL) | 63.0 ± 15.9 | 53.4 ± 15.3 | 0.13 |
| LDL cholesterol (mg/dL) | 112.0 ± 43.0 | 122.1 ± 30.0 | 0.31 |
| HOMA-IR | 2.2 ± 1.5 | 1.4 ± 1.3 | 0.23 |
| HOMA-β | 1.8 ± 1.5 | 1.0 ± 0.6 | 0.32 |
| Insulinogenic Index | 14.6 ± 20.4 | 8.2 ± 7.3 | 0.85 |
| Adipocyte IR index | 10.1 ± 11.4 | 3.5 ± 3.4 | 0.15 |

Values are the mean ± S.D.
