# Supplementary material for: Impact of Glucose Loading on Variations in CD4+ and CD8+ T Cells in Japanese Participants with or without Type 2 Diabetes
Source: Front Endocrinol (Lausanne). 2018 Mar 20;9:81. doi: 10.3389/fendo.2018.00081 (PMC5870166; doi:10.3389/fendo.2018.00081)
Supplement: Supplementary file 14 [file table_14.doc]

Table s14. Changes in the proportion of the T cell subset at 120 min after glucose loading during an OGTT in the ARB and non-ARB groups

|  | ARB | | Non-ARB | *P* value |
| --- | --- | --- | --- | --- |
| CD4^+^ (%) | | 2.57 ± 2.57 | 1.35 ± 2.12 | 0.39 |
| CD8^+^ (%) | | -2.93 ± 0.86 | -1.44 ± 1.84 | 0.14 |
| Treg (%) | | -0.73 ± 2.21 | 0.32 ± 2.00 | 0.44 |
| CD4^+^/CD8^+^ | | 0.46 ± 0.25 | 0.18 ± 0.22 | 0.06 |
| Treg/CD4^+^ | | -0.01 ± 0.02 | 0.00 ± 0.02 | 0.45 |

Values are the mean ± S.D.
