# Supplementary material for: Impact of Glucose Loading on Variations in CD4+ and CD8+ T Cells in Japanese Participants with or without Type 2 Diabetes
Source: Front Endocrinol (Lausanne). 2018 Mar 20;9:81. doi: 10.3389/fendo.2018.00081 (PMC5870166; doi:10.3389/fendo.2018.00081)
Supplement: Supplementary file 15 [file table_15.doc]

Table s15. Baseline clinical characteristics about medication use of study participants

|  | DM | NDM | *P* value |
| --- | --- | --- | --- |
| Cholesterol-lowering agents (%) | 36.8 | 23.8 | 0.37 |
| Blood pressure-lowering agents (%) | 68.4 | 57.1 | 0.46 |
